# Improving animal welfare using continuous nalbuphine infusion in a long-term rat model of sepsis

**DOI:** 10.1186/s40635-017-0137-2

**Published:** 2017-04-20

**Authors:** Victor Jeger, Mattia Arrigo, Florian F. Hildenbrand, Daniel Müller, Paulin Jirkof, Till Hauffe, Burkhardt Seifert, Margarete Arras, Donat R. Spahn, Dominique Bettex, Alain Rudiger

**Affiliations:** 1 Institute of Anaesthesiology, University of Zurich and University Hospital Zurich, Zurich, Switzerland; 2 Department of Medicine, University of Zurich and University Hospital Zurich, Zurich, Switzerland; 3 Department of Cardiology, University Heart Centre, University Hospital Zurich, Zurich, Switzerland; 4 Institute of Clinical Chemistry, University of Zurich and University Hospital Zurich, Zurich, Switzerland; 5 Department of Surgery, University of Zurich and University Hospital Zurich, Zurich, Switzerland; 6 Epidemiology, Biostatistics and Prevention Institute (EBPI), Department of Biostatistics, University of Zurich, Zurich, Switzerland

**Keywords:** Animal welfare, Analgesia, Nalbuphine, Sepsis, Rat model, Faecal peritonitis, 3R

## Abstract

**Background:**

Sepsis research relies on animal models to investigate the mechanisms of the dysregulated host response to infection. Animal welfare concerns request the use of potent analgesics for the *Refinement* of existing sepsis models, according to the 3Rs principle. Nevertheless, adequate analgesia is often missing, partly because the effects of analgesics in this particular condition are unknown. We evaluated the use of nalbuphine, an opioid with kappa agonistic and mu antagonistic effects, in rats with and without experimental sepsis.

**Methods:**

Male Wistar rats were anesthetized with isoflurane and instrumented with a venous line for drug administration. Arterial cannulation allowed for blood pressure measurements and blood sampling in short-term experiments of non-septic animals. Nalbuphine (or placebo) was administered intravenously at a dose of 1 mg/kg/h. Long-term (48 h) experiments in awake septic animals included repetitive clinical scoring with the Rat Grimace Scale and continuous heart rate monitoring by telemetry. Sepsis was induced by intraperitoneal injection of faecal slurry. Nalbuphine plasma levels were measured by liquid chromatography—high resolution mass spectrometry.

**Results:**

In anesthetized healthy animals, nalbuphine led to a significant reduction of respiratory rate, heart rate, and mean arterial pressure during short-term experiments. In awake septic animals, a continuous nalbuphine infusion did not affect heart rate but significantly improved the values of the Rat Grimace Scale. Nalbuphine plasma concentrations remained stable between 4 and 24 h of continuous infusion in septic rats.

**Conclusions:**

In anaesthetised rats, nalbuphine depresses respiratory rate, heart rate, and blood pressure. In awake animals, nalbuphine analgesia improves animal welfare during sepsis.

## Background

Sepsis is defined as life-threatening organ dysfunction caused by a dysregulated host response to infection [1, 2]. Due to the complexity of the illness, current sepsis research relies on animal models to investigate underlying mechanisms and novel therapeutic options [3].

Faecal peritonitis in rats is a widely used animal model in sepsis research [4–7]. To date, this animal model has mostly been performed without adequate analgesia, although faecal peritonitis may cause considerable suffering [8]. Animal welfare strongly requests adequate analgesia for research animals [9]. Nevertheless, adequate analgesia is often missing, partly because treatment recommendations are missing, partly because the effects of analgesics in this particular condition are unknown [3]. Evidence for appropriate analgesia in septic animals is scarce and guidelines are missing. We recently reviewed different analgesic regimens in sepsis models [10].

Nalbuphine is an opioid analgesic and acts as a kappa agonist and mu-receptor antagonist. An advantage of nalbuphine is its ceiling effect compared to pure mu agonists like morphine or fentanyl [11]. Therefore, side effects such as respiratory depression or gastrointestinal complications are less common [12, 13]. In addition, nalbuphine is not a controlled substance by the law on narcotics (in contrast to buprenorphine or morphine), which facilitates its use in laboratory animals. Compared to morphine, the analgesic potential of nalbuphine has been estimated to be at 0.8–0.9 [14].

### Objectives

Aims of this placebo-controlled study were (a) to test the short-term respiratory and cardiovascular effects of nalbuphine in anesthetized non-septic animals, (b) to investigate the behavioural and cardiovascular effects of nalbuphine in awake rats with faecal peritonitis over 48 h (compared to non-septic animals and placebo), and (c) to measure nalbuphine plasma concentrations during early and established sepsis.

## Methods

### Setting

All animal experiments were performed in the animal laboratories at the University Hospital Zurich, Switzerland. The animal model has been adapted from an established model of faecal peritonitis by the modification of fluid resuscitation and the addition of antibiotic therapy and analgesia [4, 15].

### Ethical considerations

All experimental protocols were approved by the local veterinary office (Kanton of Zurich, Switzerland, applications 95/2012 and 163/2014). Experiments are graded as class I (European category: “non-recovery”) for short-term experiments, class II (European category: “moderate”) for long-term experiments in non-septic control animals and class III (European category: “severe”) for long-term experiments in septic animals [16]. Principles of the 3Rs were implemented, and humane endpoints were applied as described below.

### Research animals

Male Wistar rats (mean weight 416 ± 76 grams, n = 45 were housed in groups of three to four animals in the local animal unit for at least 5 days prior to the experiments, with free access to food and water, a card board tube and autoclaved hay as nest material. During experiments, animals were single-housed.

### Study design

**Fig. 1:**
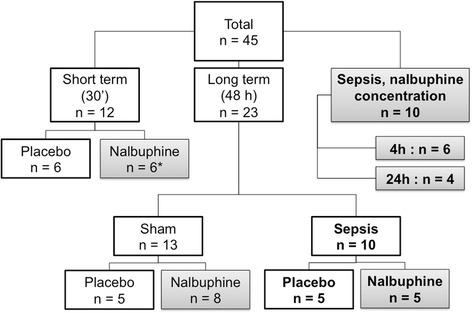
Randomization of animals to experimental groups. Animals were randomized within the short term and within the long term group. *Grey background*: groups receiving nalbuphine; *bold font*: septic groups. *Asterisk* (*): nalbuphine concentration at 15 min measured in plasma samples from short-term group

**Fig. 2:**
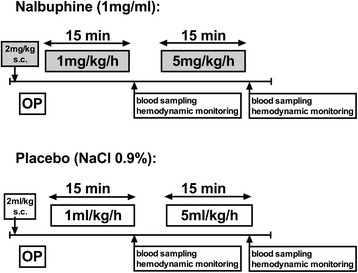
Design of short-term experiments. Animals were randomized to either nalbuphine or placebo (NaCl 0.9%) infusions. OP operation (=instrumentation). The subcutaneous administration of nalbuphine in combination with lidocaine was requested by the animal ethics committee in order to avoid post-operative pain after termination of isoflurane anaesthesia. Placebo animals received a subcutaneous injection of normal saline (2 ml/kg)

**Fig. 3:**
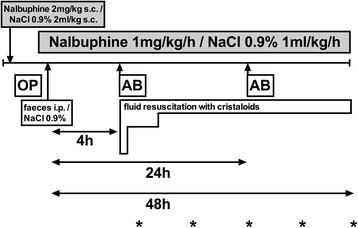
Design of long-term experiments. Animals underwent either faecal peritonitis or sham (NaCl 0.9%) i.p. injection and were subsequently randomized to either nalbuphine (1 mg/kg/h) or placebo (NaCl 0.9% 1 ml/kg/h) infusion. Asterisks indicate frequency of clinical scoring (6, 12, 24, 36, and 48 h). AB: antibiotics (ceftriaxone, 30 mg/kg i.v.); OP operation (=instrumentation). The subcutaneous administration of nalbuphine in combination with lidocaine was requested by the animal ethics committee in order to avoid post-operative pain after termination of isoflurane anaesthesia. Placebo animals received a subcutaneous injection of normal saline (2 ml/kg)

Randomized, placebo controlled animal model. Animals were either randomized to short-term (30 min) placebo (*n* = 6) and short-term nalbuphine (*n* = 6) or randomized to long-term (48 h) sham or sepsis, with or without nalbuphine (*n* = 23). Figure 1 summarises the animal numbers per group and Figs. 2 and 3 display the study design for the short- and long-term experiments.

### Nalbuphine

The content of one vial of nalbuphine (20 mg/2 ml; OrPha Swiss GmbH, Kuesnacht, Switzerland) was diluted in 18 ml of NaCl 0.9%, to obtain a solution with 1 mg/ml. The dose for this study was established in pilot experiments (*n* = 4): One septic animal benefited from a dose increment from 0.5 to 1 mg/kg/h iv, which reduced clinical pain according to the Rat Grimace Scale. However, no benefits were noted when the nalbuphine dose was further increased. Hence, the dose of 1 mg/kg/h was used in subsequent experiments.

### Experimental protocols

#### Short-term experiments (30 min)

General anaesthesia was induced by isoflurane, which was administered to the spontaneously breathing animals at a dose of 2.5% in room air (gas flow 400 ml/min). Lidocaine 1% (local pharmacy, University Hospital Zurich, Switzerland) was used at a dose of 2 ml/kg for local anaesthesia. After surgical exposure, PVC tubes (ID 0.58 mm, OD 0.96 mm; Smiths Medical Int, Kent, UK) were inserted into the right jugular vein and the right carotid artery. The fluid filled arterial line was attached to a pressure transducer (ADInstruments, Oxford, UK), which allowed blood pressure measurements and sampling of arterial blood. The transducer was connected via a bridge amplifier (ML 221, ADInstruments, Oxford, UK) to a recorder (Power Lab 4/30, ML 866, ADInstruments, Oxford, UK) and a computer. Blood pressure, heart rate, and respiration rate were recorded and analysed with specialised software (LabChart Pro 7 for Windows, ADInstruments, Oxford, UK). Blood gas analysis was performed with an Epoc blood analysis system (Epocal Inc. Ottawa, ON, Canada).

Twelve healthy animals were randomized to nalbuphine or placebo without induction of sepsis. Animals in the nalbuphine group received 2 mg/kg nalbuphine subcutaneously for analgesia after anaesthesia induction, but prior to instrumentation, the subcutaneous administration of nalbuphine in combination with lidocaine was requested by the animal ethics committee in order to avoid post-operative pain after termination of isoflurane anaesthesia. Placebo animals received a subcutaneous injection of normal saline (2 ml/kg). After instrumentation, a nalbuphine infusion was started at a dose of 1 mg/kg/h. After 15 min, the dose was increased to 5 mg/kg/h for another 15 min. After each phase, blood sampling and hemodynamic monitoring was performed (Fig. 1). All animals remained under isoflurane anaesthesia until they were euthanized by an intravenous injection of 1 ml phenobarbital (Esconarkon ad us. Vet., Streuli Pharma AG, Uznach, Switzerland) at the end of the experiment.

#### Long-term model (48 h)

Under anaesthesia as described above, two electrodes and a telemeter (TR50B, Millar, Houston, TX, USA) were implanted subcutaneously [17]. Heart rate was recorded continuously by a wireless data acquisition system (TR180 SmartPad, Millar, Houston, TX, USA). Heart rate readings were recorded with PowerLab and analysed with LabChart Pro (see Jeger et al. [10] for picture) by a blinded investigator (M.A.). Delta heart rate was calculated as heart rate at time point *x* minus heart rate at baseline.

Subsequently, a PVC tube was inserted into the right jugular vein and tunnelled subcutaneously to emerge at the nape of the neck. This line was subsequently attached to a swivel-tether system (UNO BV, Zevenaar, the Netherlands) allowing the rat to have unrestrained movement in its cage and free access to food and water (see Jeger et al. [10] for picture).

Sepsis was induced by an intraperitoneal injection of faecal slurry (*n* = 10), which contained faeces collected from several animals of the same batch, diluted in Ringer’s acetate (suspension 25%), and then filtered. After sepsis induction, isoflurane anaesthesia was stopped and the animals were transferred into their cages. Sham animals (*n* = 13) received no intra-peritoneal injection to avoid injury of abdominal organs.

Fluid resuscitation with Ringer’s acetate (Ringerfundin, B. Braun Medical AG, Sempach, Switzerland) was started four hours after peritonitis induction through the central venous line. After a fluid bolus of 20 ml/kg given over 15 min, crystalloids were infused at a rate of 10 ml/kg/h between 4 and 8 h. At 8 h, the infusion rate was reduced to 5 ml/kg/h. At 24 h, the infusion rate was further decreased to 2.5 ml/kg/h (Fig. 3).

Ceftriaxone (Rocephin, Roche Pharma, Reinach, Switzerland) 30 mg/kg was given intravenously 4 and 24 h after the septic insult [18].

Animals from both groups were randomized to nalbuphine or placebo and observed for up to 48 h (Fig. 3). Animals in the nalbuphine group received 2 mg/kg nalbuphine (Orpha Swiss GmbH, Kuesnacht, Switzerland) subcutaneously for analgesia prior to instrumentation. Placebo animals received a subcutaneous injection of normal saline (2 ml/kg). After instrumentation, a nalbuphine infusion was started at a dose of 1 mg/kg/h and continued until the end of experiment. Animals in the placebo group received no post-operative analgesia but received a continuous infusion with NaCl 0.9% as vehicle at 1 ml/kg/h.

Pain and disease severity were regularly assessed using the Rat Grimace Scale [19] and a clinical score including back arching, writhing, twitching, piloerection, and/or bloated abdomen (Table 1). The investigators of the clinical score and the Rat Grimace Scale (TH, PJ) were blinded to the treatment regimen (nalbuphine vs placebo).

**Table 1:** Score sheet to assess sepsis-induced behaviour changes

Rat grimace scale				Clinical scoring*			
Orbital tightening	0□	1□	2□	Reduced activity	0□	1□	2□
Nose/cheek flattening	0□	1□	2□	Sunken flanks	0□	1□	2□
Ear changes	0□	1□	2□	Back arching	0□	1□	2□
Whisker changes	0□	1□	2□	Piloerection	0□	1□	2□
				Bloated abdomen	0□	1□	2□
				Chromodacryorrhea (= eye discharge)	0□	1□	2□

Legend: The score sheet was adapted from [17] and www.ahwla.org.uk. *Six or more points in the clinical scoring lead to a preterm abortion of the experiment by injection of 1 ml phenobarbital i.v.

Humane endpoints (requested by the animal ethics committee) were reached if 6 or more points were obtained in the clinical score. Animals were euthanized as described above when termination criteria were fulfilled or at the end of the experiment after 48 h.

#### Nalbuphine plasma concentrations

Animals were prepared as described above. Arterial blood was sampled 15 min (*n* = 6) in short-term experiments and 4 hours (*n* = 6) and 24 h (*n* = 4) after the septic insult. These plasma samples were obtained for another study, using the same setup (data not shown). Nalbuphine was determined in plasma using liquid chromatography—high resolution mass spectrometry (LC-HRMS). To 150 μl of sample, 75 μl of methanol containing the internal standard (naltrexone, 50 μg/L) was added. After centrifugation for 10 min at 4 °C and 11,700×*g*, 80 μl were directly injected into the turbulent flow online extraction system. As mobile phases, 10 mM ammonium acetate in water + 0.1% formic acid and 10 mM ammonium acetate in methanol/acetonitrile 50/50 v/v + 0.1% formic acid were used. For online extraction, a Cyclone column (50 × 0.5 mm) and for analytical chromatography a Hypersil Gold C8 column (100 × 3mm) were used. The mass spectrometer was operated in positively heated electrospray ionization mode.

### Statistics

All results are indicated as mean ± standard deviation. Normality distribution was assumed but it could not be tested conclusively due to the small sample size of each group. Independent groups were compared using the unpaired *t* test, paired data were compared using the paired *t* test (short-term experiments). Repeated measures ANOVA were used to detect time or drug effects on heart rate over time. Missing values were replaced by the last recorded value (carrying forward) for heart rate and clinical scores. One-way ANOVA was used to determine differences between more than two groups, followed by post-hoc tests corrected by the Bonferroni method for multiple comparisons (nalbuphine plasma concentrations, cumulative clinical scores). Graph-Pad Prism 6 (GraphPad Software, La Jolla CA, USA) was used to calculate the statistics and to draw the figures.

## Results

### Short-term experiments (30 min)

During isoflurane anaesthesia, an infusion of nalbuphine at 1 mg/kg/h led to a significant reduction of respiratory rate, heart rate and mean arterial pressure (Table 2) compared to the placebo-group. Blood sampling following the higher nalbuphine dose (5 mg/kg/h) revealed lower arterial oxygen saturation and reduced pH when compared to the prior blood sampling at 1 mg/kg/h nalbuphine in the same animals.

**Table 2:** Results from short-term experiments

	Placebo		Nalbuphine			Placebo		Nalbuphine			
			1 mg/kg/h infusion		Placebo vs Nalbuphine 1 mg/kg/h			5 mg/kg/h infusion		Placebo vs Nalbuphine 5 mg/kg/h	Nalbuphine 1 mg/kg/h vs 5 mg/kg/h
	(*n* = 6)		(*n* = 6)			(*n* = 6)		(*n* = 6)			
	mean	±SD	mean	±SD	*p* value	mean	±SD	mean	±SD	*p* value	*p* value
Respiratory parameters											
Respiration rate [1/min]	55	7.7	45	4.1	*0.021*	51	9.1	41	4.7	*0.035*	0.10
pO_2_ [kPa]	8.85	1.39	7.86	2.41	0.41	9.42	2.17	6.81	0.89	*0.022*	0.19
SaO_2_ [%]	91	4.7	84	8.1	0.09	90	8.2	78	6.6	*0.017*	*0.024*
pCO_2_ [kPa]	6.13	0.68	7.72	1.28	*0.023*	6.60	1.11	8.49	0.95	*0.010*	0.07
HCO_3_ [mmol/l]	27.0	3.0	29.7	3.6	0.19	27.2	1.9	28.5	3.5	0.42	0.30
pH	7.38	0.04	7.32	0.03	*0.018*	7.35	0.06	7.26	0.03	*0.007*	*0.004*
Base excess [mmol/l]	1.9	3.4	3.6	3.3	0.40	1.6	1.6	1.5	3.7	0.94	0.08
Hemodynamic parameters											
Heart rate (1/min)	409	23.5	336	30.2	*<0.001*	408	30.4	332	40.3	*0.004*	0.58
Mean arterial pressure (mmHg)	96	14.7	78	10.3	*0.037*	89	13.1	70	6.4	*0.010*	0.09
Lactate [mmol/l]	1.0	0.3	0.9	0.2	0.22	1.1	0.3	0.9	0.1	0.20	0.71
Haematocit [%]	36	5.9	37	3.6	0.82	34	3.1	33	3.5	0.45	*0.031*

Legend: Data are shown as mean ± standard deviation (SD). All data were obtained while animals were under isoflurane anesthesia. Blood sampling was performed twice in each animal (Fig. 1). Nalbuphine 1 vs 5 mg/kg/h was compared using a paired *t* test. Nalbuphine vs placebo was compared using an unpaired t-test, as these two groups were independent. italic values indicate significance *p* < 0.05

### Long-term experiments (48 h)

**Fig. 4:**
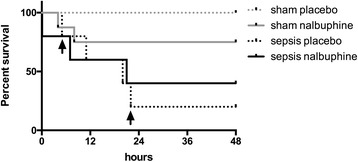
Survival curve of long-term experiments (48 h). Survival of long term experiments (48-h observation). *Arrows* indicate termination of experiments in the sepsis placebo group due to high clinical scoring

**Fig. 5:**
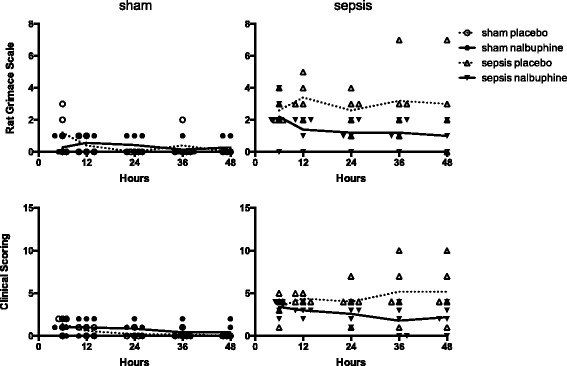
Rat Grimace Scale and clinical scoring over time. Individual scores are displayed over time. To determine the effect of nalbuphine on behaviour in sham and septic rats, cumulative scores of each animal were compared by ANOVA followed by post-hoc tests. In septic animals, nalbuphine significantly reduced the scores of the Rat Grimace Scale at 24 h (9 ± 2 vs 5 ± 3, *p* < 0.043), but not the clinical scores at 24 h (12 ± 3 vs 9 ± 2, *p* = 0.190). Due to mortality and premature termination of experiments, cumulative scores were not compared at 48 h in septic animals. In sham animals, there was no difference in cumulative scores at 24 and 48 h with and without nalbuphine. Data is shown as mean ± standard deviation

All sham animals treated with placebo survived (mortality 0%, Fig. 4). In the non-septic sham group, two out of eight animals randomized to nalbuphine died (mortality 25%; death at 4 and 8 h after the end of instrumentation). The clinical scores in the non-septic animals remained low during the entire observation period with and without nalbuphine (Fig. 5). There was no significant difference in the Rat Grimace Scale and the clinical score at 24 and 48 h between the sham placebo and sham nalbuphine groups.

In the sepsis group, three out of five animals randomized to nalbuphine died (mortality 60%; one shortly after peritonitis induction, one each at 8 and 21 h, Fig. 4). No septic animal in the nalbuphine group fulfilled the predefined termination criteria. In the septic animals randomized to placebo, two out of five died (one each at 11 and 21 h). However, two out of the three remaining animals from this group had to be prematurely euthanized at 5 and 22 h due to the high clinical scoring (>6 points) as requested by the animal ethics committee. When the cumulative scores after 24 h were compared, nalbuphine could decrease the Rat Grimace Scale at 24 h, whereas the clinical score was not significantly different (Fig. 5).

**Fig. 6:**
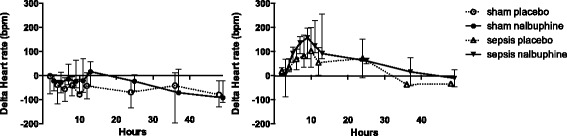
Heart rate changes over time. Delta heart rate was calculated as heart rate at time point *x* minus heart rate at baseline. Data are shown as mean ± standard deviation. Repeated measures ANOVA revealed a time effect for sham and septic animals (*p* < 0.01, each). There was no group effect or time-group interaction due to nalbuphine in sham or septic animals

Baseline heart rate for sham and septic animals were 394 ± 49 and 365 ± 50 bpm, respectively. Changes in heart rate are shown in Fig. 6. In sham animals, heart rate decreased from 394 ± 49 to 320 ± 44 bpm, *p* = 0.002 (paired *t* test) over time. In septic animals, heart rate increased after sepsis induction and started to decrease after 8 to 10 h. Heart rates were not significantly different between nalbuphine and placebo within sham or sepsis groups.

### Nalbuphine plasma concentrations

**Fig. 7:**
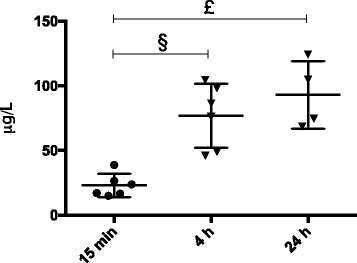
Nalbuphine plasma levels. Nalbuphine plasma levels at 15 min (sham nalbuphine, *n* = 6), 4 h (sepsis nalbuphine, *n* = 6) and 24 h (sepsis nalbuphine, *n* = 4). Samples were obtained from independent animals without repeated blood sampling (one-way ANOVA *p* < 0.001). Post-hoc test revealed significantly higher plasma levels at 4 h (§: *p* = 0.002) and 24 h (£: *p* < 0.001) vs 15 min. There was no significant difference between 4 and 24 h

Nalbuphine plasma concentration was 22.9 ± 9.0 μg/l after the combination of a s.c. nalbuphine bolus of 2 mg/kg and an intravenous infusion of 1 mg/kg/h over 15 min. Plasma concentrations in septic animals after 4 and 24 h of a continuous nalbuphine-infusion of 1 mg/kg/h were 76.7 ± 24.7 μg/l and 93.0 ± 26.2 μg/l, respectively, and significantly higher compared to blood samples drawn after 15 min (Fig. 7). However, there was no significant difference between 4 and 24 h.

## Discussion

The main findings of this study were that (a) nalbuphine led to a reduction of respiratory rate, heart rate and mean arterial pressure during general anaesthesia with isoflurane; (b) a fivefold increase of the nalbuphine dose did not worsen these variables compared to the lower dose; (c) a continuous nalbuphine infusion did not affect the behaviour of sham animals; (d) septic animals receiving nalbuphine had lower values in the Rat Grimace Scale, which could reflect reduced pain and distress perception; (e) in septic animals, continuous nalbuphine infusion had no influence on heart rate; and (f) nalbuphine plasma concentrations were stable without accumulation over time.

Analgesia during experimental sepsis is an issue of animal welfare. It corresponds to the *Refinement* of the 3R principles [20]. Consequently, legal restrictions and animal ethics get more and more demanding. In Switzerland, the introduction of the term *Animal’s Dignity* in national law requires strict obligations by the local animal committees [21]. This is even more true for level 3 animal experiments (corresponds to the European category “severe”) like awake sepsis models as described in the present study.

In awake and therefore spontaneous breathing animal models of sepsis, the use of pure mu agonists as fentanyl or morphine has major drawbacks like severe respiratory depression, prolonged gastric emptying and delayed gastrointestinal passage. Therefore, the use of mixed kappa agonists and mu antagonists like buprenorphine or nalbuphine has advantages [12]. Their ceiling effect reduces the incidence of respiratory depression and gastrointestinal side effects [13, 22]. In contrast to buprenorphine, nalbuphine is not regulated by the law on narcotics. It can therefore be ordered and stored without special restrictions, which facilitates its use in laboratory animals.

Our data on the use of nalbuphine in combination with isoflurane confirmed an earlier study in which nalbuphine plus cyclopropane induced some degree of respiratory depression [13]. At similar infusion rates as used in our study, a plateau effect on respiratory depression was observed. Only an infusion rate of 12 mg/kg/h induced a further increase of arterial PCO_2_ [13]. If nalbuphine is used in addition to inhalation anaesthesia, we suggest to increase the oxygen content in the inspiratory gas to avoid hypoxemia. Of note, excessive oxygen supply may be harmful [23]. It has been shown that nalbuphine reduces the minimal alveolar concentration (MAC) of inhalation anaesthetics which might potentiate respiratory depression [13]. In our study, animals in the placebo group did not react to painful stimuli during surgery, nor did they show a higher heart rate. This suggests, that subcutaneous nalbuphine in addition to lidocaine prior to instrumentation and the continuous infusions after venous cannulation does not add any benefit to the research animal, but puts it at risk to hypoxemia and hypercapnia. We therefore suggest to use local anaesthesia (e.g., lidocaine s.c.) in combination to inhalation anaesthetics and to withhold nalbuphine until the end of anaesthesia.

We evaluated the analgesic effects using both, clinical scoring and the Rat Grimace Scale. Recently, it has been shown that the Rat grimace scale could be used in real-time compared to the standard method of retrospective video-still analysis [24]*.* In sham animals, the analysis of cumulative scores revealed no significant effect of nalbuphine on the behaviour in comparison to placebo. This suggests that intravenous nalbuphine does not mimic the sepsis phenotype in control animals. However, 2 out of 8 animals in the sham group treated with nalbuphine died, and the cause therefore remains unclear. Potentially, hypoxia and/or hypercapnia during instrumentation may have caused the demise of these animals. In placebo treated animals, an observation over 48 h revealed a low severity in clinical scores. This supports the notion, that severity of instrumentation and single-cage housing for 48 h alone can be classified as class II (European category: “moderate”).

In septic animals, placebo-treated animals showed signs of severe illness. Two out of three animals fulfilled the predefined termination criteria and had to be euthanized. In contrast to the placebo group, septic animals receiving nalbuphine did never reach the maximum scores, which would have let to termination of the experiment. Overall, we observed lower values of the Rat Grimace Scale in the nalbuphine group compared to placebo. These effects were obtained without influencing the disease itself as suggested by the fact that sepsis-induced increase of heart rate was not blunted by nalbuphine. Therefore, we could reach our main goal of improving animal welfare by reducing distress in severe animal experiments without detecting heart rate changes over time. To our knowledge, there are no reports of nalbuphine and its effect on behaviour changes in experimental sepsis so far [10]. Further research is needed to implement robust, user-independent items for score-sheets to assess pain and distress in experimental sepsis.

Regarding plasma concentrations, a continuous intravenous infusion of 1 mg/kg/h nalbuphine over 15 min did not reach steady state drug concentrations when compared to the plasma levels at 4 h. Once a steady state was reached, our results were within the range observed by others [13]. In established sepsis (24 h), we observed stable plasma concentrations and no further accumulation. Assessment of pain in animals is difficult, even with clinical scoring systems. Therefore, patient-data may help to correlate plasma-levels with its anti-analgesic effects. Pugh et al. used a similar infusion rate as in the present study in patients after abdominal surgery with adequate analgesic action and very comparable plasma-levels (37–145 μg/L) [25].

### Limitations

The limitations of our study are the rather small numbers of animals per group. Our data are therefore underpowered to exclude that the observed beneficial effect of nalbuphine on the wellbeing of Wistar rats had an adverse effect on mortality. No repeated blood sampling was performed in long-term experiments. However, repetitive blood sampling (e.g., via tail vein under brief sedation) likely would have influenced the outcome during the 48-h mortality study. Premature termination of experiments affected the outcome. A clinical score of six had been defined as a humane endpoint by our veterinary committee. There is however scarce evidence in septic rats that this threshold corresponds to imminent death. Within the short-term experiments, the order of the low and high nalbuphine dose was always the same; therefore, a bias due to the order of the experiment cannot be excluded. Finally, we cannot provide data on possible immune system changes in sepsis due to nalbuphine. However, interactions of the immune system with opioids have mainly been reported for morphine [26, 27]. In CLP mice, buprenorphine or tramadol had some effects on white blood cells and cytokines [28, 29].

The present study has only been performed in male Wistar rats. However, sex as well as strain differences have been reported, which should be taken into consideration for the translation of our results to other setups [10, 30].

## Conclusions

If nalbuphine is used during inhalative anaesthesia with isoflurane, laboratory rats are at risk of hypoventilation with subsequent hypoxemia and hypercapnia. It might be advisable to add extra oxygen to the inspiratory gas or, even better, to withhold the nalbuphine administration until the end of the inhalative anaesthesia.

Further behavioural studies are needed to compare changes in rat models of faecal peritonitis with CLP models in mice, where more extensive research has already been done [28]. The behavioural changes and clinical effects due to sepsis in these two species are quite different and there are no studies comparing them both in a uniform setting. Especially the correlation of clinical scores with objective parameters (laboratory parameters or physiological variables) should be evaluated to determine better thresholds for humane endpoints. Furthermore, effects on inflammation/immunomodulation and organ dysfunction should be included in the assessment of analgesic treatments in sepsis. As buprenorphine is more widely used than nalbuphine, the direct comparison of both analgesics could be another focus for upcoming studies. In this context, special emphasis has to be made for comparisons of continuous iv infusion versus repeated sc injections. Buprenorphine may be more suitable for repeated sc injections due to its longer half-life compared to nalbuphine. Our results from continuous infusions can therefore not be directly translated to repeated sc injections.

In awake experimental sepsis, a continuous infusion of nalbuphine 1 mg/kg/h seems to provide sufficient analgesia in septic rats without affecting the course of the disease. Hence, nalbuphine can be recommended in such experiments of high severity as it improves animal welfare.

## Abbreviations

3R: Replacement, reduction, refinement
ANOVA: Analysis of variance
MAC: minimal alveolar concentration
